# History of the Confederate States Navy

**Published:** 1887-10

**Authors:** 


HISTORY OF THE CONFEDERATE STATES NAVY. By Col. J. T. Scharf. 824 pp. Profusely illustrated. W. H. Shepard & Co., Atlanta, Ga.

We are free to say that we have met with nothing in the shape of war literature that is equal in attractiveness to this remarkable volume.

The facts which it embodies are gathered from reliable sources and these are incorporated into the narrative with rare taste and excellent judgment. This department of Confederate history has been strangely overlooked. Col. Scharf has done, not only the South but the whole country, a signal service by his laborious research in this fresh field of historical study. The press North and South heartily endorse the history as a magnificent contribution to American annals. It is already having a rapid sale. The book, however, is only sold by subscription.

